# *Astragalus membranaceus* Extract Prevents Calcium Oxalate Crystallization and Extends Lifespan in a *Drosophila* Urolithiasis Model

**DOI:** 10.3390/life12081250

**Published:** 2022-08-16

**Authors:** Szu-Ju Chen, Sunderiya Dalanbaatar, Huey-Yi Chen, Shih-Jing Wang, Wei-Yong Lin, Po-Len Liu, Ming-Yen Tsai, Der-Cherng Chen, Yung-Hsiang Chen, Wen-Chi Chen

**Affiliations:** 1 Division of Urology, Department of Surgery, Taichung Veterans General Hospital, Taichung 40705, Taiwan; 2 Department of Family Medicine, Second State Central Hospital, Ulaanbaatar 15160, Mongolia; 3 Graduate Institute of Integrated Medicine, School of Medicine, College of Chinese Medicine, China Medical University, Taichung 40402, Taiwan; 4 Department of Obstetrics and Gynecology, Department of Medical Research, Department of Neurosurgery, Department of Urology, China Medical University Hospital, Taichung 40447, Taiwan; 5 Department of Respiratory Therapy, College of Medicine, Kaohsiung Medical University, Kaohsiung 80708, Taiwan; 6 Department of Chinese Medicine, Kaohsiung Chang Gung Memorial Hospital and Chang Gung University College of Medicine, Kaohsiung 83301, Taiwan; 7 Department of Psychology, College of Medical and Health Science, Asia University, Taichung 41354, Taiwan

**Keywords:** *Astragalus membranaceus*, *Drosophila* animal model, lifespan, traditional Chinese medicine, urolithiasis, urinary stone disease

## Abstract

Approximately 1 in 20 people develops kidney stones at some point in their life. Although the surgical removal of stones is common, the recurrence rate remains high and it is therefore important to prevent the occurrence of kidney stones. We chose *Astragalus membranaceus* (AM), which is a traditional Chinese medicine, to study the prevention of urolithiasis using a *Drosophila* model based on our previous screening of traditional Chinese herbs. Wild-type *Drosophila melanogaster* Canton-S adult fruit flies were used in this study. Ethylene glycol (EG, 0.5%) was added to food as a lithogenic agent. The positive control agent (2% potassium citrate (K-citrate)) was then compared with AM (2, 8, and 16 mg/mL). After 21 days, the fruit flies were sacrificed under carbon dioxide narcotization, and the Malpighian tubules were dissected, removed, and processed for polarized light microscopy examination to observe calcium oxalate (CaOx) crystallization. Then, the ex vivo dissolution of crystals in the Malpighian tubules was compared between K-citrate and AM. Survival analysis of the EG, K-citrate, and AM groups was also performed. Both 2% K-citrate and AM (16 mg/mL) significantly inhibited EG-induced CaOx crystal formation. Mean lifespan was significantly reduced by the administration of EG, and the results were significantly reversed in the AM (8 and 16 mg/mL) groups. However, AM extract did not directly dissolve CaOx crystals in *Drosophila* Malpighian tubules ex vivo. In conclusion, AM extract decreased the ratio of CaOx crystallization in the Malpighian tubules and significantly ameliorated EG-induced reduction of lifespan. AM prevented CaOx crystal formation in the *Drosophila* model.

## 1. Introduction

A urinary stone is a crystal concretion generally formed within the kidneys. Urinary stone disease or urolithiasis is common, painful, and associated with significant medical expenditure [1]. It is a highly prevalent urological disorder occurring in 8–19% of males and 3–5% of females worldwide [2,3]. In addition, the prevalence is increasing globally, irrespective of age, sex, and race, with rates ranging from 7% to 13% in North America, 5% to 9% in Europe, and 1% to 5% in Asia [4]. The etiology of kidney stones is multifactorial; the risk factors include age, gender, geographic variation, climate and seasonal factors, diet, fluids, occupational factors, inheritance and genetics, and numerous associated diseases (such as obesity, diabetes mellitus, hypertension, gout, and metabolic syndrome). General dietary recommendations of increased fluid, decreased salt, and moderate intake of protein have not changed. Understanding the impact of associated conditions will improve the prevention of stone disease [5]. It is associated with an increased risk of end-stage renal failure [6].

Lifestyle and dietary habits (such as high salt and protein consumption and low fluid intake) can induce urinary metabolic abnormalities, which undoubtedly play an important role in the risk of urinary stone disease [5,7,8]. The symptoms of urolithiasis include flank pain (the pain can be quite severe) and blood in the urine (hematuria). Calcium oxalate (CaOx) constitutes the majority of stones formed in the human kidney. Nonsteroidal anti-inflammatory drugs were the preferred analgesic option for renal colic patients presenting to the emergency department. Medical treatment is crucial for patients suffering from urolithiasis to palliate pain, enhance spontaneous stone passage, and prevent stone regrowth and recurrences [9]. Unfortunately, since the 1980s, when potassium citrate (K-citrate) was introduced, new drugs have not been developed for stone prevention, perhaps because the long observation time needed to demonstrate efficacy discourages researchers from undertaking clinical studies [10]. In addition, extracorporeal shock wave lithotripsy (ESWL) and minimally invasive approaches such as percutaneous nephrolithotomy and retrograde intrarenal surgery are considered as the mainstay for the treatment of kidney stones. More recently, laser devices have been used for lithotripsy during these procedures [11]. Although surgical treatment of stone disease has greatly advanced, there have been no improvements in the prevention strategies. Recently, Wu et al. evaluated the outcomes of patients with active surveillance or prophylactic intervention for asymptomatic kidney stones and found that patients who underwent prophylactic intervention had lower risks of stone-related events and future intervention [12]. Therefore, there is an urgent need to identify the potential antilithic effects of medicines to prevent stone disease.

*Astragalus membranaceus* (Fisch.) Bunge (AM; Chinese name: Huangqi/Huang-Chi) is a herb of traditional Chinese medicine (TCM) that has been used for thousands of years in Taiwan, China, and East Asia to treat kidney disease [13]. AM is a perennial herbaceous plant; *Astragalus* belongs to the *Leguminosae* family. In TCM, the root of AM is believed to tonify Qi, induce diuresis, relieve edema, and nourish blood. It may also replenish body fluids and relieve pain [13]. Our earlier screening of 80 herbs of TCM using an animal model of *Drosophila* indicated that AM has a potential antilithic effect for urolithiasis [14]. However, screening studies of TCM are limited; further, there is a new study model to dissolve CaOx crystals ex vivo [15]. In this study, we aimed to investigate the antilithic effect of different concentrations of AM extract by increasing the number of animals in the study along with the addition of this new model.

## 2. Materials and Methods

### 2.1. Fruit Fly Stocks and Rearing Conditions

We used wild-type adult male fruit flies, *Drosophila melanogaster* Canton-S, in this study. About 200 fruit flies (3 independent experiments were performed) in each group were bred in plastic vials containing standard fly medium at 25 °C and 60% humidity, with a 12 h light−dark cycle [16]. The formula of the standard fly medium consisted of 0.67 g of agar, 2.17 g of yeast, 1.31 g of sugar, and 6.66 g of corn powder with the addition of water to a final amount of 100 mL. The solution was put in a microwave to boil. Then, 1.33 mL of 99% alcohol and 0.34 g of β-hydroxybenzoic acid methylester were added after cooling to 65 °C. Next, 10 ml of the medium was decanted into a 50 mL test tube and stored in a 4 °C freezer after the medium returned to room temperature (ready for use only within a 21-day interval).

### 2.2. Lithogenesis of Drosophila

We used 0.5% EG as lithogenic agent added to the nutritional medium. The 2% K-citrate (Gentle Pharma Co. Ltd., Yunlin, Taiwan) was used as a positive control. The extracts of AM were purchased from Sun Ten Pharmaceutical Co., Ltd. (Taichung, Taiwan). Since a herbal extract of TCM, AM, was used for the study, the high-performance liquid chromatography (HPLC) analysis for the main components’ fingerprints was provided as a reference for the quality control of the medicinal herb. The quality control of AM with composition ratio of each component and elucidation of the mixture components is shown in Supplementary Materials Figures S1–S4. The study groups of crystal formation by AM were 2, 8, and 16 mg/mL. All groups of fruit flies were fed with 0.5% EG from the beginning to the end of experiment (exception: control group). After 3 weeks, the fruit flies were sacrificed under carbon dioxide (CO_2_) narcotization, and the Malpighian tubules were microscopically dissected, removed, and processed for polarized light microscopy (Olympus BX51) examination. The different degrees (Grade 0, 1, 2, and 3) of CaOx crystal deposition in the Malpighian tubules were rated. Each blinded specimen was evaluated by three investigators who assessed crystal formation using a crystal score of 0 = none, 1 = weak, 2 = moderate, and 3 = strong [16]. The number of each grade crystal formation among the groups was calculated and labelled as N_0_ × 0, N_1_ × 1, N_2_ × 2, and N_3_ × 3. The average score of crystal formation in each group was calculated as (N_0_ × 0 + N_1_ × 1 + N_2_ × 2 + N_3_ × 3)/total number of fruit flies.

### 2.3. Lifespan Assay

To conduct the lifespan experiment, new sprung fruit flies were collected under light by CO_2_ using anesthesia. Foam plugs were used by researchers because the food vials were used horizontally to prevent weaker fruit flies accidentally being adhered to food or cotton plugs. About 200 fruit flies (3 independent experiments were performed) in each group. Each survivor in the vials needed to be counted and dead fruit flies were taken out of the vials daily. Survival was compared and checked for significance with log-rank tests. Lifespan curves were from pooled counts of a huge number of vials. An adult needs nearly 2 kg of food and water per day, totaling 2000 kcal or 8400 kJ [17]. The package insert (Off-Label Use) of the TCM concentrated herbal extract recommends the directions for adults are 1.2 to 3 g daily. We chose 2 g/day as a standard human dosage to calculate the fruit fly dosage. Human dosage: 2 g (daily drug weight)/2000 g (daily human food weight) = fruit fly dosage: 0.1% (*w*/*w*; in fly medium). Based on our previous experience [14], we used 2×, 8×, and 16× (2, 8, and 16 mg/mL, respectively) to evaluate the potential dose-dependent effects of AM on lithogenesis and lifespan tests.

### 2.4. CaOx Crystal Dissolution Assay Ex Vivo

Methods of the dissolution of CaOx crystal ex vivo was modified from Fan et al. [18]. Briefly, intact Malpighian tubules were dissected in phosphate buffered saline (PBS) under dissection microscope and transferred onto a slide from 3 weeks after lithogenesis. We added 100 μL PBS solution to completely cover the Malpighian tubule tissue and subjected it to live-imaging under polarized light with an Olympus BX51 optical microscope without a coverslip. Images were taken every 40 min to 2 h and overnight (0 min, 40 min, 80 min, 120 min, and ~16 h). The total area of Malpighian tubules was photographed in both light and dark fields. The grades of crystal dissolution were evaluated in each interval. Grading of the final crystal dissolution was as 0: no dissolution, 1: partial dissolution, and 2: complete dissolution. Three independent experiments were performed in each group. The crystal dissolution rate was calculated by the sum of the grade divided by the total number of Malpighian tubules in each group. The rate of dissolution was calculated in time series.

### 2.5. Statistical Analyses

One-way analysis of variance was used to analyze overall differences between the groups. Bonferroni correction was applied for all multiple comparisons. For the ratio between two lifespan curves, we determined the *p* value in the log-rank test. All statistics were produced using the Statistical Package for Social Sciences (SPSS for Windows, release 28.0, SPSS Inc., Chicago, IL, USA). Statistically significant difference is defined as *p* value below 0.05.

## 3. Results

### 3.1. Crystal Formation

**Figure 1:**
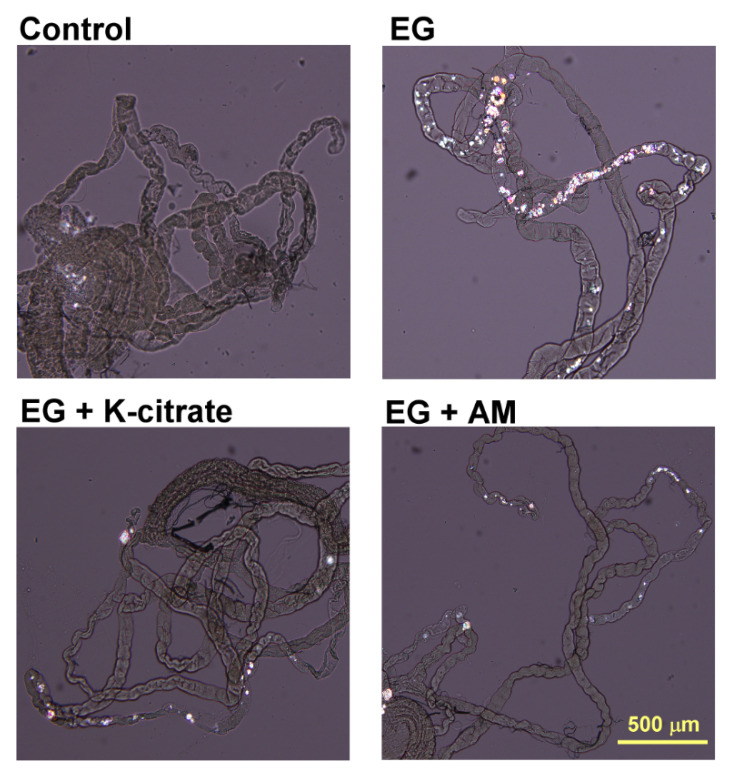
Effects of K-citrate (2%) and AM (16 mg/mL) on EG-induced crystal deposition in the Malpighian tubules of *Drosophila*. The images show representative polarized microscopy for the *Drosophila* with 0.5% EG-induced crystal formation in Malpighian tubules. Scale bar = 500 μm.

**Figure 2:**
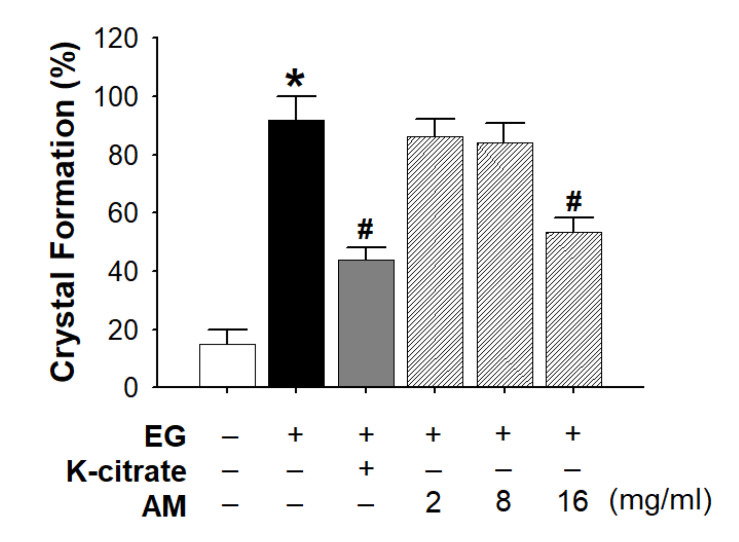
Rates of CaOx crystal formation in the in vivo study. Crystal formation in 0.5% EG, 2% K-citrate, and 2, 8, and 16 mg/mL AM-treated *Drosophila* (*n* ≅ 200 for each group; three independent experiments were performed in each group). Data are expressed as mean ± S.D. * *p* < 0.05, compared to the control. ^#^ *p* < 0.05, compared to the 0.5% EG-treated group.

The crystallization grade was measured after completion of 3 weeks of breeding. Figure 1 shows a typical morphological pattern of EG-induced CaOx crystal deposition and K-citrate/AM-treated groups in the Malpighian tubules. Table 1 shows the number and average grades of CaOx crystallization in the control, 0.5% EG, 2% K-citrate, and 2, 8, and 16 mg/mL AM groups. K-citrate had a protective effect on CaOx crystallization (43.9 ± 4.1%), which reduced the grades of crystallization compared to the EG group (91.9 ± 8.2%). Only high-dose AM (16 mg/mL) reduced crystallization (53.5 ± 4.8%), similar to K-citrate (Figure 2).

### 3.2. Lifespan Assay

**Figure 3:**
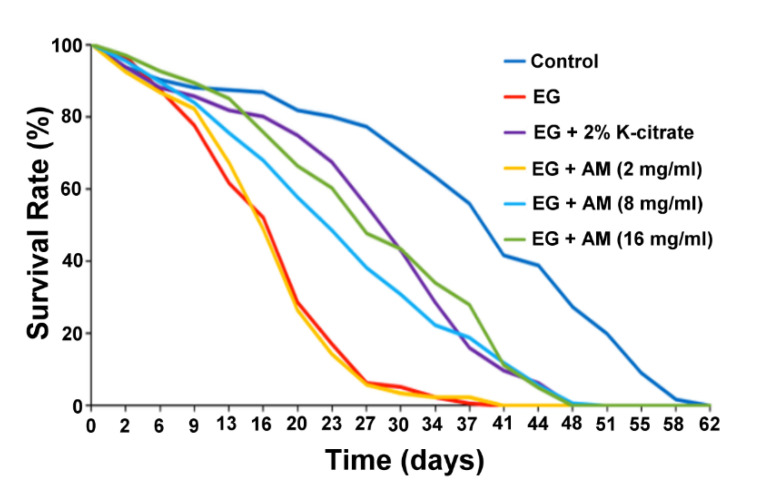
Survival curve of lifespan for control, 0.5% EG, EG + 2% K-citrate, and EG + 2, 8, and 16 mg/mL AM-treated *Drosophila* (*n* ≅ 200 for each group; three independent experiments were performed in each group). For ratio between two lifespan curves, we determined the *p* value in the log-rank test.

**Figure 4:**
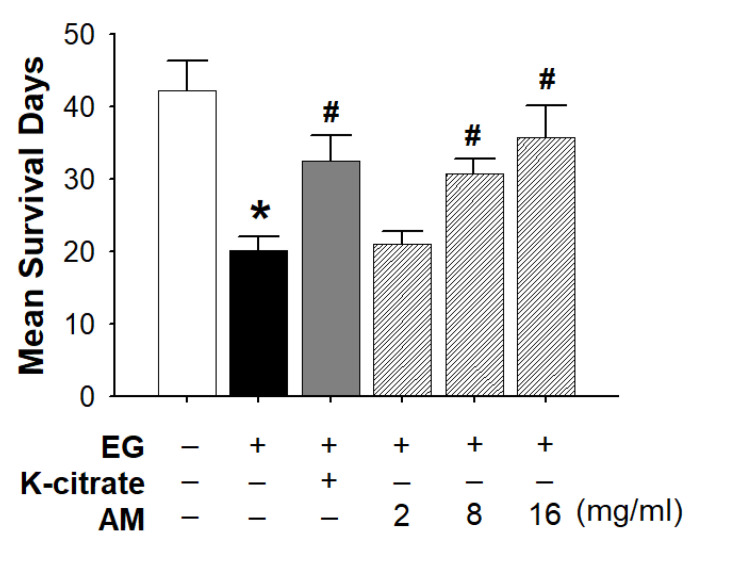
Effects of K-citrate and AM extract on lifespan. Effect of EG, EG + K-citrate, and EG + 2, 8, and 16 mg/mL AM on lifespan of *Drosophila* (*n* ≅ 200 for each group; three independent experiments were performed in each group). Data are expressed as mean ± S.D. * *p* < 0.05 compared to the control; ^#^ *p* < 0.05 compared to the 0.5% EG-treated group.

To test whether the effects of AM were associated with a decreased mortality rate under treatment with the lithogenic agent EG, the lifespan of *Drosophila* was measured after the flies’ food was supplemented with K-citrate and various concentrations of AM (Table 2). Figure 3 shows the survival survey results of each group. EG has a toxic effect on the lifespan of *Drosophila*, with the shortest mean survival time. The K-citrate and AM (8 and 16 mg/mL, respectively) groups had a protective effect with longer mean survival days than the EG group (Figure 4).

### 3.3. Ex Vivo Dissolution of CaOx Crystals in Malphigian Tubules

**Figure 5:**
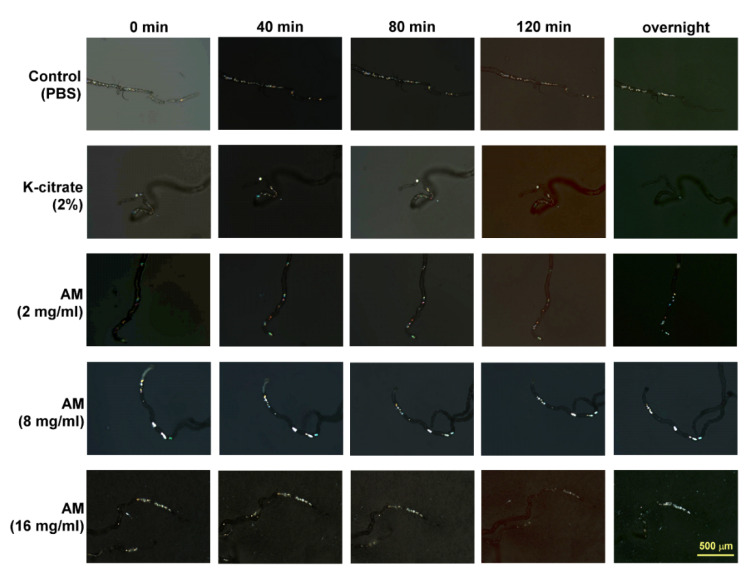
K-citrate, but not AM, extract directly dissolves CaOx crystals in *Drosophila* Malpighian tubules ex vivo. Represented images of CaOx crystal dissolution in intact Malpighian tubules treated with 2% K-citrate and AM (2, 8, and 16 mg/mL) solutions ex vivo at different time points.

**Figure 6:**
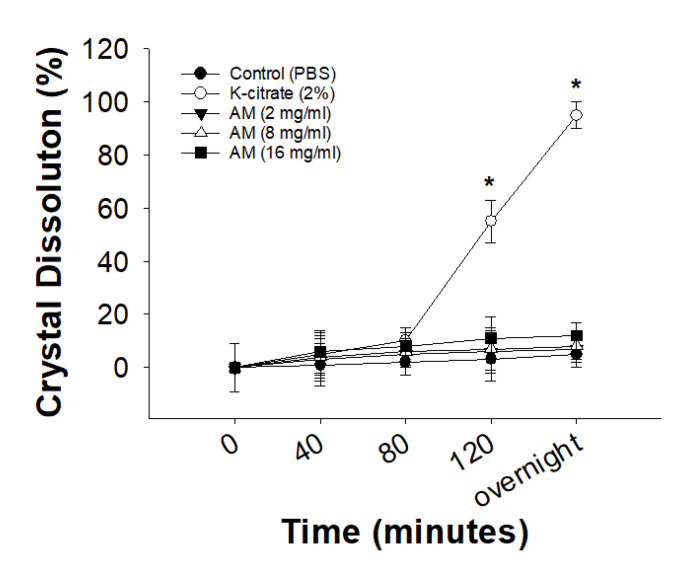
K-citrate, but not AM, extract directly dissolves CaOx crystals in *Drosophila* Malpighian tubules ex vivo. Wild-type *Drosophila* was reared in fly food containing 0.5% EG for 3 weeks. Intact Malpighian tubules were dissected from *Drosophila* and treated with PBS, 2% K-citrate, and AM solutions. CaOx crystal dissolution was monitored using live-imaging. Renal CaOx crystal dissolution rate was calculated. Data are expressed as mean ± S.D. (three independent experiments were performed in each group). * *p* < 0.05 compared to the control.

To explore the mechanism through which AM inhibits CaOx crystal formation, we examined the dissolution of CaOx crystals in Malpighian tubules ex vivo. The rate of dissolution in each group was calculated at different times. K-citrate directly dissolved the CaOx crystals in the Malpighian tubules ex vivo in a time-dependent manner. During the observation period, no CaOx crystals were excreted from the Malpighian tubule. Almost 100% of the CaOx crystals were dissolved in a 2% K-citrate solution after overnight (~16 h) treatment. In contrast, PBS and AM extracts had no effect on the dissolution of CaOx crystals preformed in the Malpighian tubule of *Drosophila* (Figure 5 and Figure 6).

## 4. Discussion

In this study, we examined the antilithogenic effects of different concentrations of AM extract and compared them with those of K-citrate. The inhibitory effect of AM, comparable to that of K-citrate, was observed only at a high dose of 16 mg/mL. The control group had a mean and maximum lifespan of 42.3 ± 4.1 and 62 days, respectively. The mean lifespan was significantly reduced by the administration of EG, while the administration of AM resulted in decreased CaOx crystal formation and increased lifespan. Regarding ex vivo dissolution, K-citrate had the highest rate of dissolution, while AM extract had no direct effect on the dissolution of CaOx crystals.

Despite advances in diagnosis and treatment, urolithiasis is still a disease with high prevalence and recurrence. The recurrence rate of urinary tract stones is approximately 10% per year. Half of the patients experienced recurrence within 10 years. Recurrence has been reported to occur more than once in 80% of the patients. Therefore, it is important to prevent the development of urolithiasis [19].

Landry et al. used a fruit fly model to study the homolog of Slc26a6 (a nephrolithiasis-related gene involved in oxalate transportation) related to *Drosophila* (Slc26a5/6 and dPrestin), which found that thiosulfate decreased CaOx stone formation in Malpighian tubules [20]. This result indicates that thiosulfate or oxalate mimics may act as competitive therapeutic inhibitors for the crystallization of CaOx. Fan et al. used fruit flies to perform RNAi knockdown of nephrolithiasis-related genes in the principal cells of Malpighian tubules, and studied the effects of antilithic agents on CaOx crystal formation [18]. Therefore, genetic studies of stone disease in fruit flies may have future potential [21,22,23]. The pathogenesis of CaOx formation is a multistep process and essentially includes nucleation, crystal growth, crystal aggregation, and crystal retention. Many inorganic and organic substances are known to modulate stone formation. A comprehensive account of the mechanisms of renal stone formation does not yet exist, and the role of promoters/inhibitors in CaOx crystallization is still unclear [24]. Nevertheless, we speculate that these nephrolithiasis-related genes associated with calcium and oxalate transportation or metabolization might possibly be involved in the molecular mechanisms of AM treatment for kidney stones.

Chen et al. first observed the formation of CaOx crystals in Malpighian tubules within weeks after the addition of lithogenic agents in fly food and directly examined them under polarized light microscopy [8]. Since then, *Drosophila* models have been used to screen potential antilithic traditional Chinese medicinal plants with 80 candidate herbs [14]. Wu et al. reported that the use of certain tested herbs of TCM, including *Salviae miltiorrhizae*, *Paeonia lactiflora*, *Carthami flos*, and *Scutellaria baicalensis* resulted in a crystal formation rate of approximately 0.0%. However, the actual mechanism causing this remains unknown, and related studies lack the adequate number of animals required to establish statistical significance [25,26]. Thus, *Salviae miltiorrhizae* and *Carthami flos* were further studied using rat animal models, as well as with additional clinical analyses [14,27,28,29,30]. *Drosophila* models, therefore, provide a fast-breeding and large population, which assists in the screening of potential antilithic drugs. Chen et al. developed a new observation method in which a whole fruit fly can be viewed using micro-computerized tomography, which makes this model more beneficial [31].

AM is a well-known “Qi-tonifying” or adaptogenic herb used in TCM. Its Chinese name, “Huangqi/Huang-Chi”, means “yellow leader”. It is a long yellow tap root and is deservedly popular because it helps the body in a variety of ways. AM was listed in the first order of the traditional herb drug book, *Ben Cao Bei Yao* (“Essentials of materia medica”), as one of the most important drugs among herbs of TCM. It has been prescribed for centuries for general debilitation and chronic illnesses, and to increase overall vitality. It offers several health benefits, such as boosting the immune system [32], promoting natural urination, supporting normal body fluids, and benefiting the respiratory system. AM is often used in liver and blood tonics. It possesses antiviral, antioxidant, antibacterial, and detoxifying properties. According to a meta-analysis of studies from China, AM was found to have renal protective effects in 1804 diabetic patients with nephropathy [33,34]. In TCM [35,36], *Astragalus* is used either alone or in combination with other herbs in various doses and forms [37]. For example, AM combined with Chuan Xiong (*Ligusticum chuanxiong* Hort., a major component of tetramethylpyrazine) was widely used in TCM for the treatment of abdominal pain and cardiovascular disease [38].

Recently, Chung et al. reported in the journal *Nature* that hydroxycitric acid (HCA), a component of the extract from the fruit *Garcinia cambogia*, can dissolve CaOx crystals in vitro [39]. This tropical fruit found in Southeast Asia, which is rich in HCA, is traditionally used for cooking purposes in India and may be a potential antilithogenic herb. Fan et al. and Chen et al. further studied HCA to analyze its preventative effects on CaOx formation in *Drosophila* models [15,18,40]. However, the ex vivo effect of HCA on the dissolution of CaOx crystals differed from that of both these groups when compared with that of K-citrate. Fan et al. reported that 1% HCA completely dissolved CaOx crystals (induced by sodium oxalate) within 2 h in an ex vivo study [15,18]. Chen et al. reported that the effect of HCA was only 39% for in vivo dissolution of CaOx (induced by EG) in *Drosophila* [40]. Our results indicated that K-citrate was the most effective agent for the prevention of CaOx crystal formation in the *Drosophila* model. In contrast, most of the studies focus on the antioxidative and anti-inflammatory effects of AM. The main ingredients of AM include polysaccharides, astragalosides, saponins, and flavonoids, with strong effects on scavenging oxidative free radicals and anti-inflammation [41]. It has been reported in a clinical study that herbs (containing AM) of TCM could protect against ESWL-induced renal damage [42]. However, to the best of our knowledge, none of the main components of AM has been reported to have the same ability to dissolve CaOx crystals as HCA.

Nevertheless, *Astragalus* polysaccharides (APS) have been shown to inhibit the adhesion and promote the endocytosis of CaOx dihydrate nanocrystals to damaged renal proximal tubular epithelial cells [43]. APS could inhibit CaOx monohydrate growth, induce CaOx dihydrate formation, and increase the absolute zeta potential of the crystals to inhibit crystal aggregation [44]. Moreover, after being repaired by APS, the amount of adherent crystals on the cell surface decreased, but the amount of endocytic crystals increased [45]. Thus, these results suggest that APS may serve as potential drugs for preventing kidney stones. On the other hand, AM is a major medicinal herb that has been commonly used in many herbal formulations in the practice of TCM to treat a wide variety of diseases and body disorders, or marketed as life-prolonging extracts for human use. The major components of AM are polysaccharides, flavonoids, and saponins. Pharmacological research indicates that some of the extract components of AM can increase telomerase activity, and has various antioxidative and anti-inflammatory effects. Hence, targeting these pathological changes could reverse aging and treat age-associated diseases [46].

One of the advantages of using *Drosophila* models is their lifespan [47,48]. Chemical agents that are potentially hazardous to human health can be rapidly tested through lifespan studies because toxic agents may shorten life cycles. *Drosophila* has a short life cycle of approximately 8–9 weeks and is easy to breed in large numbers over a short period of time [48,49]. Therefore, fruit flies are an ideal model for studying potentially toxic agents. Melamine (sometimes illegally added to food products to increase their apparent protein content) and ractopamine (an animal feed additive used to promote leanness and increase food conversion efficiency) have been reported to have potentially hazardous effects on humans in Taiwan [50]. Chen et al. used a *Drosophila* model to study their effects on toxicity. Melamine significantly reduced the lifespan of *Drosophila* in a dose-dependent manner [19], while ractopamine (10 ppb added to food) significantly reduced the lifespan of fruit flies [50]. Lifespan studies can also be used to test other agents by following the aforementioned method [48,51].

The disadvantages of using a fruit fly model include the difficulty of studying the mechanisms of tubular fluid formation and the absence of serum or urine biochemistry [52]. Further, this invertebrate lacks many solid organs, including the kidneys. However, Malpighian tubules of the fruit fly have a similar genetic composition, physiology, and anatomy to those of the human kidney [22]. Miller et al. proposed that dietary manipulation, environmental alteration, and genetic variations affect stone formation and can be rapidly observed and quantified in *Drosophila* within a few days [22].

## 5. Conclusions

In summary, we demonstrated that TCM AM extract efficiently prevented CaOx crystal formation in the Malpighian tubule of *Drosophila* in vivo, but the AM extract did not directly dissolve or excrete CaOx crystals from the Malpighian tubule ex vivo. Since urolithiasis is still a disease with high recurrence, these data suggest that AM can be used as a prophylactic intervention in bodies that have the tendency to form crystals. Further clinical studies are warranted to confirm its effects on the prevention of lithiasis.

## Figures and Tables

**Table 1:** Overall CaOx crystal formation in each group of *Drosophila* (*n* ≅ 200).

Group	Total	Grade 0	Grade 1	Grade 2	Grade 3	Average Score
Control	170	144	26	0	0	0.15
EG	173	14	64	65	30	1.64 *
EG + 2% K-citrate	155	87	42	19	7	0.65 ^#^
EG + AM (2 mg/mL)	166	23	54	62	27	1.56
EG + AM (8 mg/mL)	168	28	62	52	26	1.45
EG + AM (16 mg/mL)	170	79	49	32	10	0.84 ^#^

CaOx: calcium oxalate, EG: ethylene glycol (0.5%), K-citrate: potassium citrate, AM: *Astragalus membranaceus*. * *p* < 0.05, compared to the control. ^#^ *p* < 0.05, compared to the EG-treated group.

**Table 2:** Lifespan assay in each group of *Drosophila* (*n* ≅ 200).

Group	Days Until Last Fly Died	Median Survival Days	Mean Survival Days
Control	62	40	42.3 ± 4.1
EG	41	18	20.2 ± 1.9 *
EG + 2% K-citrate	48	28	32.6 ± 3.4
EG + AM (2 mg/mL)	41	17	21.1 ± 1.8
EG + AM (8 mg/mL)	48	22	30.8 ± 2.1 ^#^
EG + AM (16 mg/mL)	51	27	35.7 ± 4.5 ^#^

EG: ethylene glycol (0.5%), K-citrate: potassium citrate, AM: *Astragalus membranaceus*. * *p* < 0.05, compared to the control. ^#^ *p* < 0.05, compared to the EG-treated group.

## Data Availability

Data will be provided on request.

## Supplementary Materials

The following supporting information can be downloaded at: https://www.mdpi.com/article/10.3390/life12081250/s1, Figure S1. HPLC analysis for *Astragalus membranaceus* extract. Figure S2. 2D-HPLC chromatogram of *Astragalus membranaceus* extract analyzed at 250 nm by photodiode array detector. Figure S3. 2D-HPLC chromatogram of *Astragalus membranaceus* extract analyzed by evaporative light scattering detector. Figure S4. 3D-HPLC chromatogram of *Astragalus membranaceus* extract.
