# Supplementary material for: Astragalus membranaceus Extract Prevents Calcium Oxalate Crystallization and Extends Lifespan in a Drosophila Urolithiasis Model
Source: Life (Basel). 2022 Aug 16;12(8):1250. doi: 10.3390/life12081250 (PMC9409928; doi:10.3390/life12081250)
Supplement: Supplementary file 1 [file life-12-01250-s001.zip › life-1803527-supplementary.pdf]

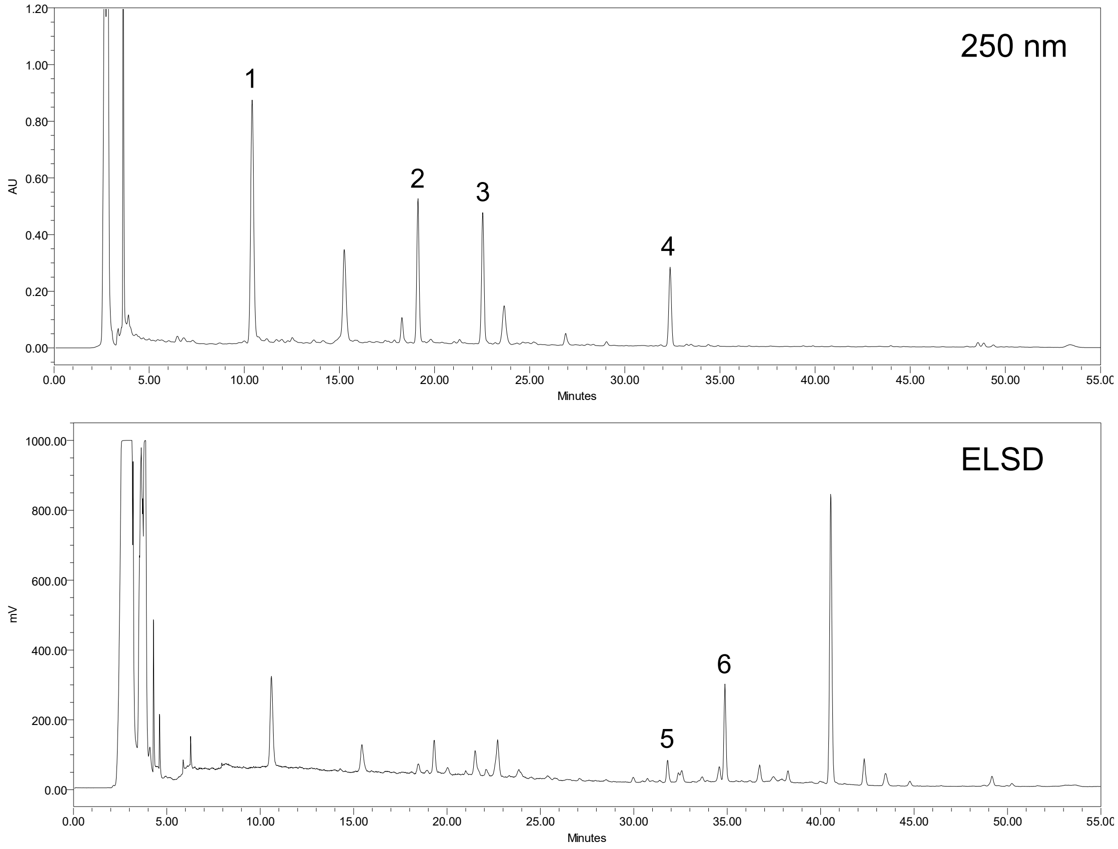

#	Name	Retention time (min)	Assay (mg/g)	Detection
1	Calycosin 7-*O*-β-D-glucoside	10.42	0.345	250 nm
2	Ononin	19.13	0.161	250 nm
3	Calycosin	22.54	0.108	250 nm
4	Formononetin	32.38	0.053	250 nm
5	Astragaloside IV	31.81	0.115	ELSD
6	Astragaloside II	34.88	0.409	ELSD

Figure S1. HPLC analysis for *Astragalus membranaceus* extract.

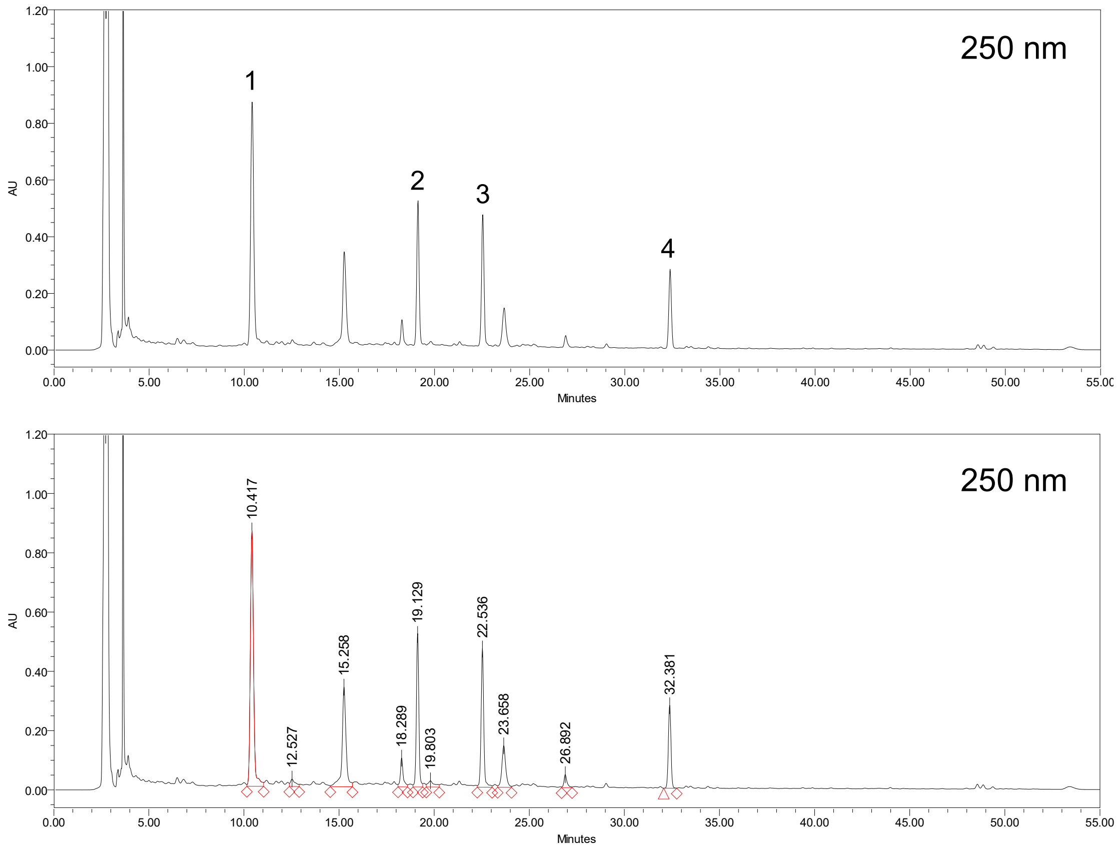

#	Name	Retention Time (min)	Area	% Area	Height	Int Type
1	Calycosin 7-*O*-β-D-glucoside	10.417	10266885	32.67	862316	VV
		12.527	440411	1.40	24731	VV
		15.258	4848909	15.43	337635	VV
		18.289	1010298	3.21	97592	VV
2	Ononin	19.129	4661005	14.83	517530	VV
		19.803	493484	1.57	20988	VV
3	Calycosin	22.536	4536924	14.43	468068	VV
		23.658	2009115	6.39	140493	VV
		26.892	485318	1.54	43511	VV
4	Formononetin	32.381	2678304	8.52	279294	BV

Figure S2. 2D-HPLC chromatogram of *Astragalus membranaceus* extract analyzed at 250 nm by photodiode array detector.

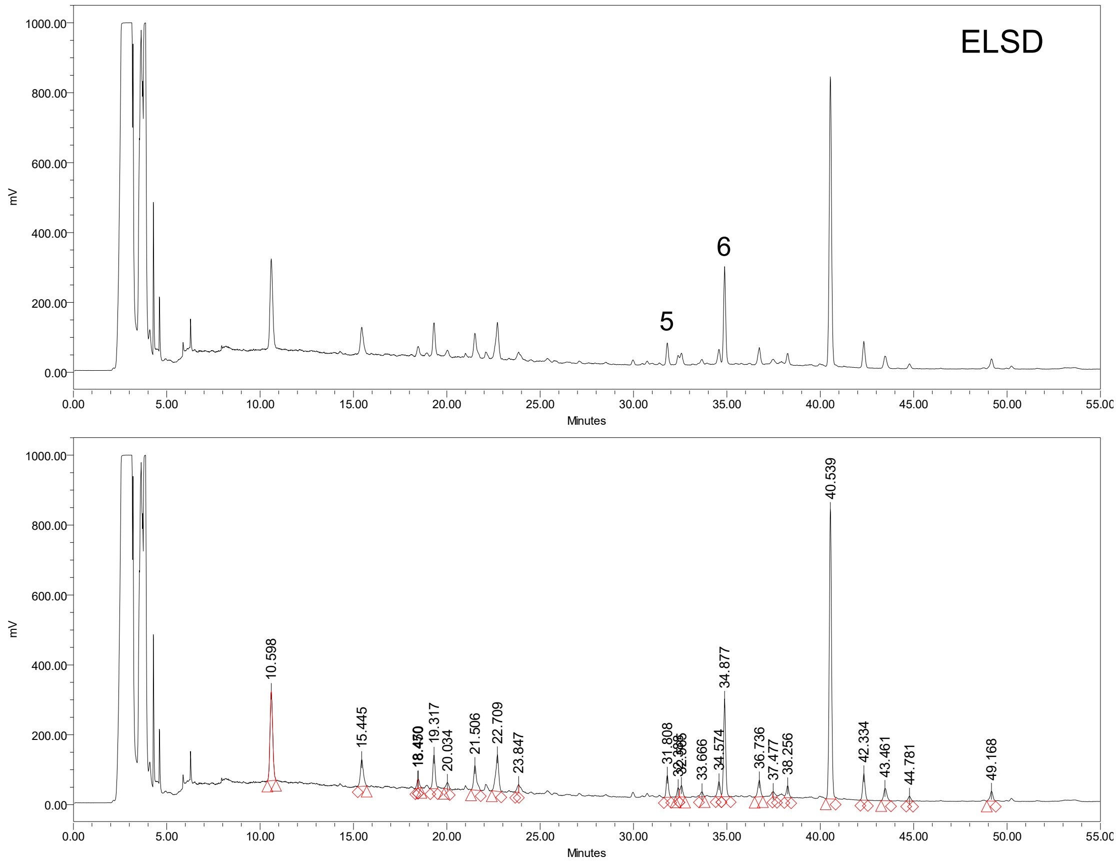

#	Name	Retention Time (min)	Area	% Area	Height	Int Type
1		10.598	2572287	12.65	256731	BB
2		15.445	867152	4.27	76994	VB
3		18.450	111571	0.55	25967	VV
4		18.470	130802	0.64	26509	VB
5		19.317	845656	4.16	95663	VV
6		20.034	189006	0.93	18785	BV
7		21.506	778282	3.83	70502	BV
8		22.709	1186365	5.84	104301	BV
9		23.847	146593	0.72	22514	VV
10	Astragaloside IV	31.808	525710	2.59	63689	VV
11		32.388	197805	0.97	26224	BV
12		32.565	323210	1.59	32574	VB
13		33.666	143634	0.71	14170	VB
14		34.574	405970	2.00	43217	VV
15	Astragaloside II	34.877	2215897	10.90	278858	VV
16		36.736	472879	2.33	48432	BB
17		37.477	148316	0.73	15830	VV
18		38.256	331681	1.63	33997	VV
19		40.539	7198956	35.41	831368	BV
20		42.334	672861	3.31	76129	VV
21		43.461	412914	2.03	35093	BV
22		44.781	142256	0.70	14197	VV
23		49.168	309852	1.52	28062	BV

Figure S3. 2D-HPLC chromatogram of *Astragalus membranaceus* extract analyzed by evaporative light scattering detector.

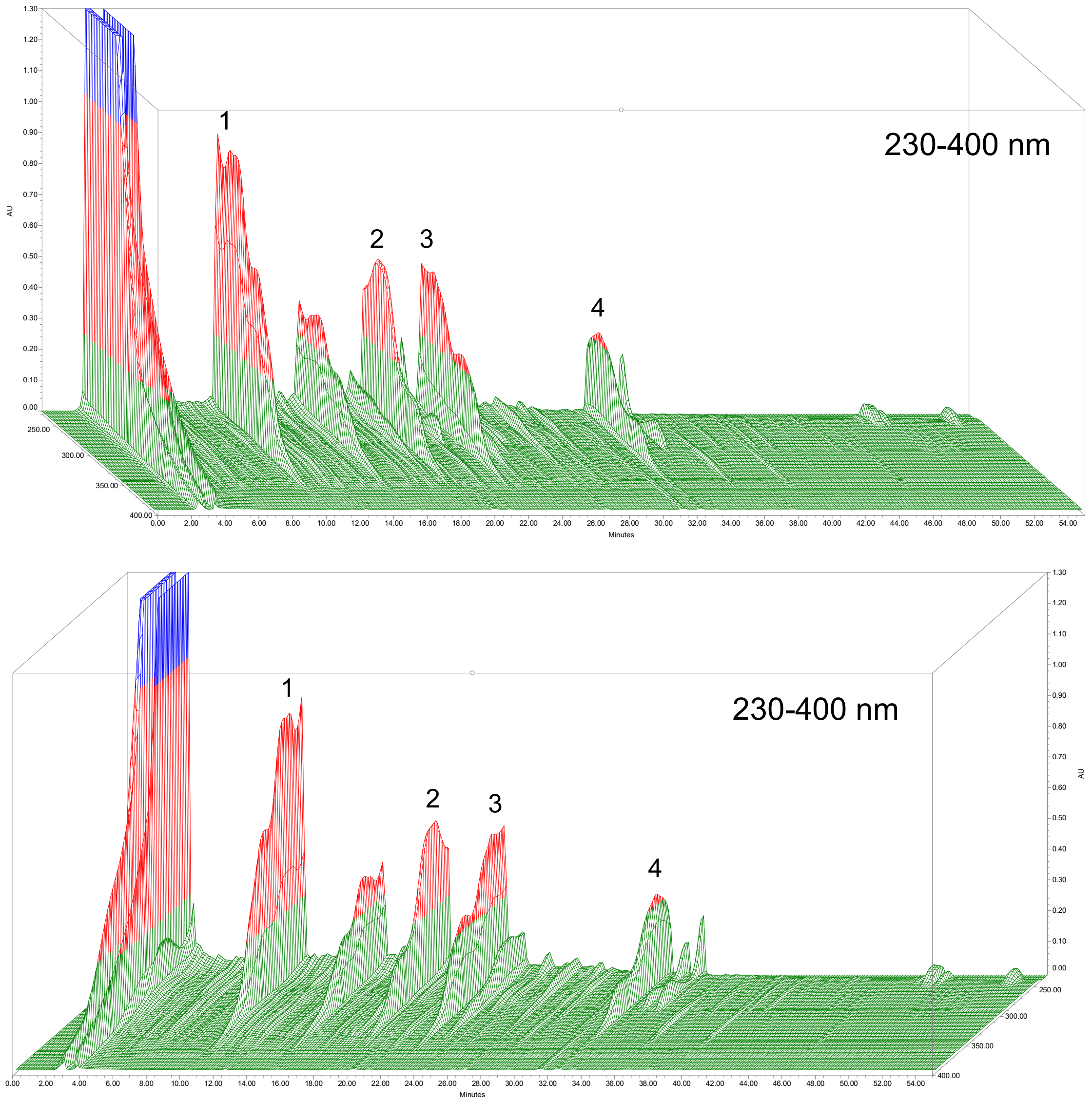

1 Calycosin 7-*O*-β-D-glucoside
2 Ononin
3 Calycosin
4 Formononetin

Figure S4. 3D-HPLC chromatogram of *Astragalus membranaceus* extract.
